# miR-130a acts as a potential diagnostic biomarker and promotes gastric cancer migration, invasion and proliferation by targeting RUNX3

**DOI:** 10.3892/or.2015.4099

**Published:** 2015-07-01

**Authors:** HONG JIANG, WEI-WEI YU, LU-LU WANG, YANG PENG

**Affiliations:** 1 Department of Geriatrics, Tongji Hospital, Tongji Medical College, Huazhong University of Science and Technology, Wuhan, Hubei 430030, P.R. China; 2 Department of Gastroenterology and Hepatology, The Second Affiliated Hospital of Chongqing Medical University, Chongqing 400010, P.R. China

**Keywords:** miR-130a, gastric cancer, diagnosis, prognosis, RUNX3, tumorigenesis

## Abstract

MicroRNAs (miRNAs) are abnormally expressed in various types of cancer. miR-130a expression and function in gastric cancer has yet to be elucidated. The aim of the present study was to identify the miR-130a expression and function in gastric cancer. miR-130a expression was examined in gastric cancer cell lines and tissues by RT-qPCR. The diagnostic and prognostic significance of miR-130a in gastric cancer was analyzed by receiver-operating characteristic (ROC) curve and Kaplan-Meier analysis. miR130a expression was identified and the diagnostic significance in the serum of gastric cancer patients and healthy controls was analyzed using RT-qPCR and ROC curves, respectively. A target gene for miR-130a was identified using luciferase reporter assays, and gastric cancer tumorigenesis ability was examined by 3-(4,5-dimethylthazol-2-yl)-2,5-diphenyltetrazolium bromide (MTT) and Transwell assays. The results showed that miR-130a was upregulated in gastric cancer. The low-miR-130a group had significantly improved overall survival compared to the high-miR-130a group. Furthermore, the expression of miR-130a in plasma in gastric cancer patients was upregulated and diagnostic value for gastric cancer of miR-130a is more effective than the tumor markers carcinoembryonic antigen (CEA) and CA-199. miR-130a directly targeted runt-related transcription factor 3 (RUNX3) and promoted gastric cancer tumorigenesis by targeting RUNX3. miR-130a may therefore be a useful marker for the diagnosis and prognosis of gastric cancer. Additionally, miR-130a was identified as an oncogene that promotes gastric cancer tumorigenesis by targeting RUNX3.

## Introduction

Gastric cancer is a common disease worldwide and the second most frequent cause of cancer-associated mortality, affecting approximately one million individuals annually (1). Of 880,000 people diagnosed with gastric cancer in 2000, approximately 650,000 (74%) succumbed to the disease (2). The genesis and progression of human gastric cancer is thought to be crucially influenced by genetic and epigenetic alterations, including the activation of oncogenes and the inactivation of tumor-suppressor genes (3). Oncogenes and tumor-suppressor genes have always been regulated by microRNAs (miRNAs).

miRNAs are non-coding RNA molecules ~21–23 nucleo tides long that regulate gene expression at the post-transcriptional level (4–6). miRNA expression profiling analyses have revealed a global dysregulation of mature miRNA levels in primary human tumors compared to normal tissues (7,8). miRNAs act as novel oncogenes or tumor-suppressor genes (9,10) and it has been shown that alterations in microRNA expression correlate highly with the progression and prognosis of human tumors (11,12). Furthermore, a number of differentially expressed miRNAs derived from circulation are used as potential biomarkers in various types of cancer, including liver, prostate, lymphoma and ovarian cancer (13–16). Thus, focusing on miRNAs in gastric cancer resulted in insight into the diagnosis and treatment of this disease.

An increasing body of evidence indicates miR-130a is differentially expressed in various tumors. miR-130a was overexpressed in adult T-cell leukemia (ATL), basal cell carcinoma and esophageal cancer tissue, but underexpressed in bladder and ovarian cancer, and glioblastoma (17–22). However, few studies have focused on miR-130a expression and its function in gastric cancer. Xu *et al* found that miR-130a directly inhibited the expression of tumor-suppressor gene runt-related transcription factor 3 (RUNX3) (23). RUNX3, a member of the family of transcription factors that contain the runt domain, is located at human chromosome 1p36 and was identified as a tumor-suppressor in breast, bladder and lung cancer (24–26). Previous findings showed that, RUNX3 was identified as a pivotal tumor-suppressor in gastric cancer (27), and a loss or substantial decrease in RUNX3 expression may be causally associated with gastric cancer, as it correlates with differentiation, lymph node metastasis and poor prognosis of this disease (28). Thus, miR-130a may act as an oncogene in gastric cancer by targeting RUNX3. Therefore, further systemic delineation of miR-130a expression and function in gastric cancer is needed.

## Materials and methods

### Human tissue specimens and cell lines

The present study utilized fresh tissues, including 41 human gastric cancer samples and 41 samples of adjacent normal mucosal tissues derived from 41 patients who underwent surgery at the Second Affiliated Hospital of Chongqing Medical University (Chongqing, China) between 2010 and 2011. The present study was conducted according to the 'Biomedical Research Involving Human Ethics Review (Tentative)' regulation of the Ministry of Health and the Declaration of Helsinki on Ethical Principles for Medical Research Involving Human Subjects. All the samples were obtained with the informed consent of the patients, and the experiments were approved by the Institutional Review Board of the Second Affiliated Hospital of Chongqing Medical University (Chongqing, China). All the participants provided written informed consent to participate in the present study.

The SGC-7901, HGC-27, AGS, MKN45 and N87 cell lines were obtained from the American Type Culture Collection (ATCC; Manassas, VA, USA), and the GES-1 cell line was purchased from the Type Culture Collection of the Chinese Academy of Sciences (Shanghai, China). The cell lines were cultured in RPMI-1640 (HyClone, Logan, UT, USA) supplemented with 10% fetal bovine serum (FBS) and were incubated at 37°C with 5% CO_2_.

### Serum collection

Whole blood (2 ml) from the gastric cancer patients and healthy controls was collected in regular tubes and immediately processed to prevent contamination by cellular nucleic acids. Blood samples were centrifuged at 2,000 rpm for 10 min at room temperature, and then the upper supernatant, which was the serum sample, was transferred to new RNase-free collection tubes, respectively, and stored at −80°C for further processing.

### Detection of CEA and CA-199

Values for carcinoembryonic antigen (CEA) and carbohydrate antigen 199 (CA-199) levels in the serum of the gastric cancer patients and healthy controls were determined at the Clinical Laboratory of the Second Affiliated Hospital of Chongqing Medical University (Chongqing, China).

### Primers, RNA isolation and miRNA detection

The primers for miR-338-3p and U6 were produced using the miScript Primer Assay kit (Qiagen, Dusseldorf, Germany). The sequences of the miRNAs used in the present study were as follows: miR-130a, CAGUGCAAUGUUAAAAGGGCAU; and U6, CGCAAGGAUGACACGCAAAUUCGUGAAGCGUUCCAUAUUUUU. The reverse primers were also used in the reverse transcription step. Total miRNA was extracted from the cultured cells, human tissue specimens and serum sample using RNAiso for small RNA (Takara Bio, Otsu, Japan) according to the manufacturer's instructions. Poly(A) tails were added to miR-338 and U6 with the miRNA Reaction Buffer Mix, and then cDNA was produced from 5 ng of total RNA using the miRNA PrimeScript RT Enzyme Mix (both from Takara Bio). RT-qPCR was performed in a CFX96™ Real-Time PCR Detection System (Bio-Rad, Hercules, CA, USA) with SYBR^®^ Premix Ex *Taq*™ II (Takara Bio). The PCR conditions used were 95°C for 30 sec, followed by 40 cycles of 95°C for 5 sec, and 60°C for 30 sec. The data were normalized against the U6 snRNA. After amplification, a melting curve analysis was performed to confirm the specificity of the products.

Expression levels of the miRNAs were calculated by cycle threshold (Ct) values with SDS 2.0 software (Applied Biosystems, Foster City, CA, USA). The concentrations from serum, tissues or cell lines samples were normalized using the 2^−ΔΔCt^ method relative to U6 small nuclear RNA (RNU6B). The value of ΔCt was calculated by subtracting the Ct values of RNU6B from the Ct values of the miRNAs of interest in the present study. The values of ΔΔCt were then calculated by subtracting the ΔCt of the control samples from the ΔCt of the cancer samples. The change in gene expression was calculated using the equation 2^−ΔΔCt^.

### Oligonucleotide transfection

miR-130a mimics, inhibitor and cont-miR were produced by Sangon Biotechnology (Sangon, Shanghai, China), and co-transfections were performed with Lipofectamine 2000 (Invitrogen-Life Technologies, Carlsbad, CA, USA). Twenty-four hours after transfection, the cells were plated for hte proliferation, migration and invasion assays. The cells were collected for RNA and protein analyses 48 h after transfection.

### pcDNA expression plasmids and plasmid transfection

The ORF sequences of RUNX3 were amplified from genomic DNA isolated from the AGS cell line and were then subcloned into the GV230 vector (GeneChem Corporation, Shanghai, China). The plasmid was transfected into AGS cells using Lipofectamine 2000. Twenty-four hours after transfection, the cells were used for a rescue experiment.

### Luciferase reporter assay

A Psicheck™-2 Dual-Luciferase miRNA target expression vector was used for the 3′UTR luciferase assays (Sangon Biotechnology). The target gene of miRNA-130a was selected based on the online microRNA target database, http://www.microrna.org/microrna/home.do. The primer sequences used for the wild-type 3′UTR of RUNX3 were: forward, 5′-CCGCCCTGGTGGACTCCT-3′ and reverse, 5′-CCTTCCACACATCTCAGAGTTATAT-3′. Since there was one binding site in the RUNX3 3′UTR, primer sequences were designed for the mutant 3′UTR as follows: forward, 5′-GTGGAAACTGTGGCGCGCCATCGTTTGCTTGGTGTTTG-3′ and reverse, 5′-GCAAACGATAGTGCA AAGCAGTTTCCACCCAGCTCCAT-3′. For the luciferase assay, Lipofectamine 2000 was used to co-transfect MKN45 cells with the miR-130a mimics and Psicheck™-2 Dual-Luciferase miRNA target expression vectors containing wild-type or mutant target sequences. The Dual-Luciferase Assay (Promega, Madison, WI, USA) was used to measure the firefly luciferase activity 18 h after transfection, and the results were normalized against the *Renilla* luciferase. Each reporter plasmid was transfected at least three times (on different days), and each sample was assayed in triplicate.

### Cell viability assays

The transfected cells were seeded in 96-well plates at a density of 1×10^4^ cells/well. A 3-(4,5-dimethyl-thazol-2-yl)-2,5-diphenyltetrazolium bromide (MTT) solution (20 ml of 5 mg/ml MTT) was added to the cultures (for a total volume of 250 *µ*l) and incubated for 4 h at 37°C. Following the removal of the culture medium, the remaining crystals were dissolved in dimethylsulfoxide (DMSO), and the absorbance at 570 nm was measured.

### Migration and invasion assays

For the Transwell migration assays, 1×10^4^ cells were plated in the top chamber with a non-coated membrane (24-well insert; 8-mm pore size; BD Biosciences, Franklin Lakes, NJ, USA). For the invasion assays, 2×10^5^ cells were plated in the top chamber with a Matrigel-coated membrane (24-well insert; 8 mm pore size; BD Biosciences). For the two assays, the cells were plated in a serum-free medium, and medium supplemented with 10% serum was used as a chemoattractant in the lower chamber. The cells were incubated for 16 h at 37°C and 5% CO_2_ in a tissue culture incubator. After 16 h, the non-migrated/non-invading cells were removed from the upper sides of the Transwell membrane filter inserts using cotton-tipped swabs. The migrated/invaded cells on the lower sides of the inserts were stained with Giemsa, and the cells were counted.

### Antibodies and immunoblotting

Antibodies against RUNX3 and Smad4 were purchased from Abcam (Cambridge, UK). Antibody against GAPDH was purchased from Santa Cruz Biotechnology, Inc. (Santa Cruz, CA, USA). HRP-conjugated goat anti-rabbit IgG was purchased from Santa Cruz Biotechnology, Inc.. The total protein was extracted from the transfected cells and gastric cancer tissues using RIPA lysis buffer (Beyotime, China) according to the manufacturer's instructions. After the whole-cell protein extracts were quantified using the BCA protein assay, equivalent amounts of cell lysates were resolved by 10% SDS polyacrylamide gel electrophoresis, and were transferred onto a polyvinylidene fluoride membrane, which was then blocked in 5% non-fat milk in TBST for 1 h at 4°C. The blots were then incubated with primary antibodies. After incubation with HRP-conjugated secondary antibodies, the protein bands were visualized using an enhanced chemiluminescence reagent (Millipore, Billerica, MA, USA). The following antibody dilutions were used: anti-RUNX and anti-Smad4, 1:1,500; and HRP-conjugated IgG, 1:7,000.

### Statistical analysis

SPSS 17.0 software was used for the statistical analysis. The data are presented as the means ± standard deviation (SD). Group comparisons were performed using the Student's t-test. The relationships between miR-130a expression in the serum of gastric cancer patients and healthy control were analyzed using the non-parametric Mann-Whitney U test. Receiver-operating characteristic (ROC) curves and the area under the ROC curve (AUC) were used to assess the feasibility of serum and tissue miRNA as a diagnostic tool for detecting gastric cancer. For disease progression, the Kaplan-Meier (log-rank test) analysis was performed. The Spearman's rank test was used to evaluate the relationships among the relative expression levels of miR-130a and RUNX3 in gastric cancer tissues. The relationships, in the AGS cell line with the forced expression of miR-130a or cont-miR and with or without RUNX3 restoration, were analyzed using the one-way ANOVA Dunnett's test. Differences were considered to indicate a statistically significant result when P<0.05.

## Results

### miR-130a is upregulated in gastric cancer tissues and cell lines

**Figure 1:**
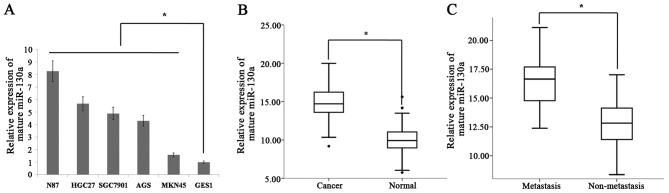
miR-130a is upregulated in gastric cancer tissues and cell lines. (A) miR-130a expression was detected in gastric cancer cell lines (SGC7901, HGC27, AGS, MKN45 and N87) and a normal gastric mucosa cell line, GES1. Data are shown as the mean ± SD (n=3) in the cell lines, ^*^p=0.021. (B) The expression level of mature miR-130a in gastric cancer (n=41) or adjacent normal mucosal tissues (n=41) was determined by RT-qPCR analysis. Data are shown separately in human samples, ^*^p=0.018. (C) Mature miR-130a expression levels in metastatic (n=25) and non-metastatic (n=16) gastric cancers. Data are shown separately in human samples, ^*^p=0.009.

To investigate the miR-130a expression levels in human gastric cancer cell lines, we monitored its expression in several cancer cell lines (SGC7901, HGC27, AGS, MKN45 and N87) and a normal gastric mucosal cell line (GES1). We found that miR-130a expression was increased in gastric cancer cell lines compared to the normal gastric mucosal cells (Fig. 1A). To further examine the role of miR-130a in human gastric cancer development, we detected the levels of its expression in 41 cases of human gastric cancer and 41 cases adjacent normal mucosa tissues. The RT-qPCR analysis revealed that, the levels of miR-130a expression were significantly increased in tumor tissues compared to that of the adjacent normal mucosal tissues (Fig. 1B). To determine whether miR-130a expression was associated with gastric cancer metastasis, we examined the miR-130a expression levels in 41 archived primary gastric tumors. These tumors were divided into two groups: in one group tumors were resected from 25 patients with lymph node metastasis, while in the second group the tumors were resected from 16 patients without metastasis. The RT-qPCR analysis revealed that, the miR-130a expression levels were significantly higher in the patients with metastasis than in those without metastasis.

### Diagnostic and prognostic significance of miR-130a in gastric cancer

**Figure 2:**
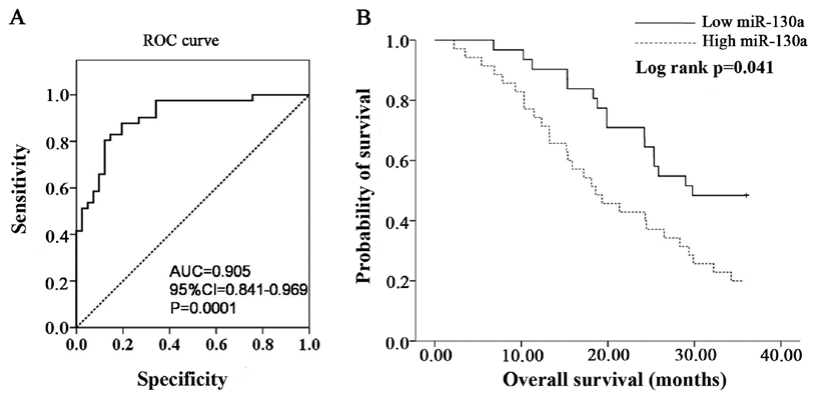
Diagnostic and prognostic significance of miR-130a is shown in gastric cancer. (A) ROC curve analysis showing performance of miR-130a expression to discriminate between malignant and non-malignant tissue samples. (B) Kaplan-Meier analysis for overall survival based on miR-130a expression. ROC, receiver-operating characteristic.

Clinical information for the 41 patients is shown in Table I. Receiver-operating characteristic (ROC) curve analyses were performed to evaluate the ability of miR-130a expression to discriminate between normal and tumor cases using tissue samples. According to ROC curve, miR-130a had the best sensitivity and specificity when the miR-130a Ct value was 11.13 (YI=0.634), and an AUC of 0.905 (P=0.0001; 95% CI, 0.841–0.969) (Fig. 2A) was obtained, suggesting that miR-130a expression discriminated between malignant and non-malignant samples and may therefore be used as a diagnostic marker for gastric cancer. To determine whether the levels of miR-130a in tumor tissues correlate with the survival of the gastric cancer patients, the patients were divided into low-miR-130a (expression Ct value <11.13) and high-miR-130a (expression Ct value ≥11.13) groups and a Kaplan-Meier survival analysis was performed. The results of the Kaplan-Meier analysis revealed that the low-miR-130a group had a significantly improved overall survival compared to the high-miR-130a group (Fig. 2B).

### miR130a expression and diagnostic significance in the serum of gastric cancer patients and healthy controls

**Figure 3:**
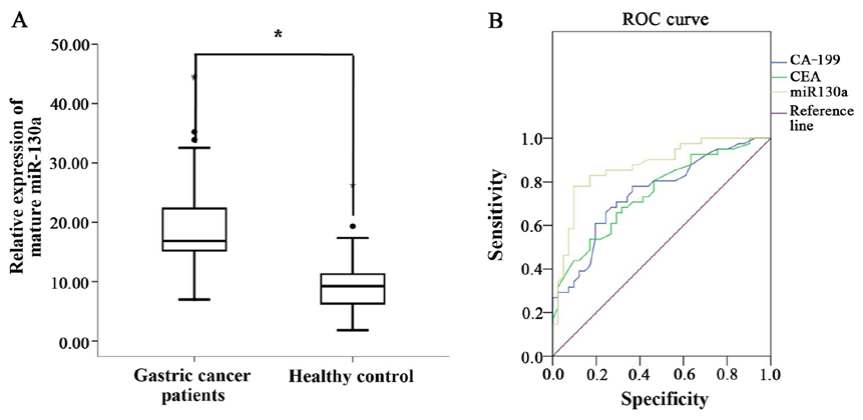
miR130a expression and diagnostic significance is shown in the serum of gastric cancer patients and healthy controls. (A) The expression level of mature miR-130a in gastric cancer patients (n=41) or healthy patients (n=41) was determined by RT-qPCR analysis. Data are shown separately in human serum samples, ^*^p=0.0041. (B) ROC analysis curve of CEA, CA-199 and miR-130a expression in the serum of the gastric cancer patients and healthy controls. ROC, receiver-operating characteristic; CEA, carcinoembryonic antigen.

Clinicopathological characteristics for the gastric cancer patients are shown in Table I. miR-130a expression was detected in the serum of gastric cancer patients and healthy controls. We also found that the expression of miR-130a in plasma in 41 gastric cancer patients was significantly higher than that from the healthy controls (Fig. 3A). To further understand the significance of the diagnostic value between miR-130a and traditional tumor markers CEA and CA-199, a ROC analysis of CEA, CA-199 and miR-130a was performed. Regarding the area under the ROC curve, miR-130a had the highest value at 0.870, while values for CEA and CA-199 were at 0.749 and 0.750, respectively (Fig. 3B and Table II). Our findings showed that the diagnostic value of miR-130a was more effective than that for tumor markers CEA and CA-199 in the serum of gastric cancer patients and healthy controls.

### miR-130a directly targets RUNX3

**Figure 4:**
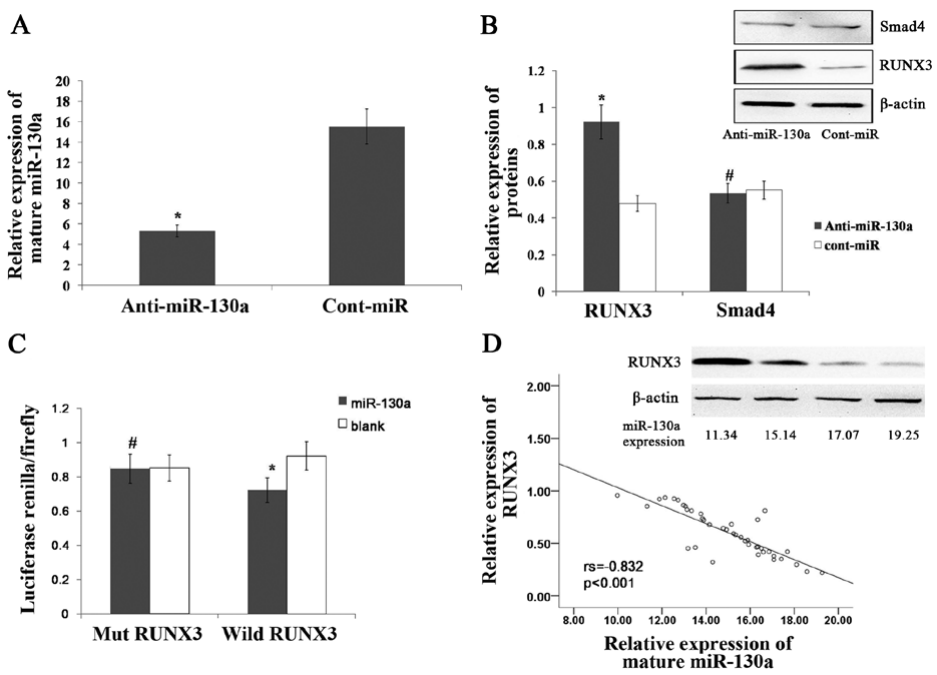
miR-130a directly targets RUNX3. (A) miR-130a expression was detected in the AGS gastric cell line after transfecting anti-miR-130a and cont-miR. ^*^P=0.0018 compared to the controls. (B) Smad4 and RUNX3 expression was examined by western blotting in the AGS gastric cell line following transfection with anti-miR-130a and cont-miR. ^*^P=0.031 and ^#^p=0.322 compared to the controls. (C) The relative luciferase activity was analyzed after the reporter plasmids were co-transfected with miR-130a mimics or control mimics into the AGS cell lines. ^*^P=0.019 and ^#^p=0.462 compared to the controls. (D) RUNX3 protein expression was examined by western blotting in gastric cancer tissues and the correlation between miR-130a and RUNX3 protein expression is shown. RUNX3, runt-related transcription factor 3.

We predicted 26 targets for miR-130a using the following prediction tools: miRanda (http://www.microrna.org), TargetScan (http://www.targetscan.org/) and miRDB (mirdb.org/miRDB/). In those 26 targets, we found some genes associated with tumors, such as RUNX3, MET, Smad4 and KLF4 (Table III). Recent findings showed that, RUNX3 and Smad4, which function as tumor-suppressor genes, are associated with gastric cancer (29–31). To determine whether miR-130a targets RUNX3 and Smad4, we examined RUNX3 and Smad4 expression in the AGS gastric cell line following transfection with miR-130a inhibitor. Compared with the expression of cont-miR, miR-130a was significantly downregulated in the AGS cell line following transfection with the miR-130a inhibitor (Fig. 4A), and we found that RUNX3 expression was significantly increased in miR-130a-low-expressing gastric cancer cell lines, although Smad4 expression was not increased (Fig. 4B). Our findings showed that RUNX3 may be the target gene for miR-130a. To confirm that miR-130a directly targeted RUNX3, we performed luciferase reporter assays to examine whether miR-130a interacts directly with RUNX3. We found that the co-transfection of miR-130a and the wild-type RUNX3 3′UTR caused a significant decrease in luciferase expression when compared with the controls. However, the co-transfection of miR-130a and the mutant RUNX3 3′UTR did not cause a decrease in luciferase expression (Fig. 4C). The RUNX3 expression was also detected in 41 gastric cancer tissues by western blotting, and we found that the expression of miR-130a was inversely correlated with that of RUNX3 (Fig. 4D). These results suggested that miR-130a directly targets RUNX3.

### miR-130a promotes gastric cancer cell migration, invasion and proliferation by targeting RUNX3

**Figure 5:**
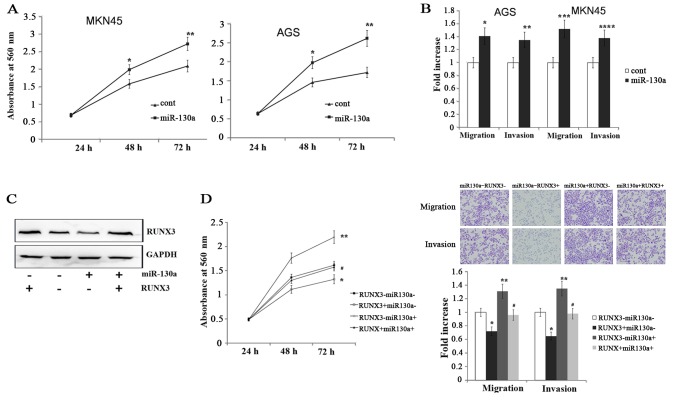
miR-130a promotes gastric cancer migration, invasion and proliferation by targeting RUNX3. (A) Cell proliferation is shown after transfection with miR-130a mimics or cont-miR in the AGS and MKN45 gastric cancer cell lines. ^*^P=0.031 and ^**^p=0.022 for the AGS cell line and ^*^p=0.031 and ^**^p=0.015 for the MKN45 cell line compared to the controls. (B) The migration and invasion of AGS and MKN45 cell lines were exhibited following transfection with miR-130a mimics or cont-miR: ^*^p=0.0018, ^**^p=0.0032, ^***^p=0.0011 and ^****^p=0.012 compared to the controls. (C) Immunoblot analysis of RUNX3 expression in AGS cells transfected with miR-130a mimics or cont-miR with or without RUNX3 restoration. (D) The tumorigenic qualities of AGS cells were detected following transfection with miR-130a or cont-miR and with or without RUNX3 restoration. Cell proliferation is shown in the left panel: ^*^p=0.0128, ^**^p=0.0022 and ^#^p=0.312. Gastric cancer migration and invasion are shown in right panel: Migration ^*^p=0.0328, ^**^p=0.0122, and ^#^p=0.215; and invasion ^*^p=0.0328, ^**^p=0.0125 and ^#^p=0.415 compared to controls.

To determine the functional significance of miR-130a in gastric cancer, we transfected the AGS and MKN45 gastric cancer cell lines with miR-130a mimics. We found that the cells with a forced expression of miR-130a significantly increased proliferation compared with the cells with a forced expression of cont-miR (Fig. 5A). Transwell migration and Matrigel invasion assays demonstrated that miR-130a significantly increased the migration and invasion of AGS and MKN45 cells (Fig. 5B). To confirm whether miR-130a promotes gastric cancer migration, invasion and proliferation by targeting RUNX3, we forced the expression of miR-130a in AGS and MKN45 cell lines along with a construct containing the RUNX3 coding sequence but lacking the 3′UTR of the RUNX3 mRNA. As a result, this construct yielded a RUNX3 mRNA that was resistant to miR-130a. The restoration of RUNX3 expression was confirmed through an immunoblot analysis (Fig. 5C). We found that the gastric cancer cell migration, invasion and proliferation were completely restored in the AGS cell line with a forced miR-130a expression and RUNX3 restoration (Fig. 5D). Therefore, miR-130a regulated gastric cancer cell migration, invasion and proliferation by targeting RUNX3.

## Discussion

In the present study, we did examined miR-130a expression in gastric cancer cell lines and detected miR-130a expression in gastric cancer tissues, and found that miR-130a expression was significantly upregulated in gastric cancer cell lines and tissues. Furthermore, miR-130a expression was significantly higher in the patients with metastasis than in the patients without metastasis. The results show that miR-130a as an oncogene was overexpressed in gastric cancer and was associated with metastasis. miRNAs possess several features that make them attractive candidates as new prognostic biomarkers and powerful tools for the early diagnosis of cancer (32,33). ROC and Kaplan-Meier analysis were than performed to determine the miR-130a diagnostic and prognostic potential in gastric cancer. Our findings show that miR-130a can be used as a diagnostic marker for gastric cancer and low levels of miR-130a were significantly associated with an extended overall survival of gastric cancer patients. Thus, miR-130a serves as a molecular diagnostic and prognostic marker for gastric cancer patients.

Screening for early gastric cancer potentially reduces mortality of the disease (34–36). Since the currently known tumor markers have the limitation of low sensitivity and specificity (37), diagnosis of gastric cancer is performed using a gastroscopic biopsy sample and histology specified by WHO criteria. Although gastroscopic screening for gastric cancer is currently the most reliable screening tool, it is expensive and unsuitable as a first-line examination due to the invasive nature. Thus, identification of novel non-invasive biomarkers for tumor detection is imperative. Previous findings have shown that some miRNAs have potential diagnostic value in different types of cancer (19,38,39). In the present study, we found that miR-130a expression in serum was overexpressed in gastric cancer patients than healthy controls. Compared with the diagnostic value of CEA, CA-199 and miR-130a individually, miR-130a had the highest value at 0.870 on the area under the ROC curve. miR-130a is sufficiently reliable for the diagnosis of gastric cancer. Thus, miR-130a acts as a potential circulating diagnostic biomarker for gastric cancer.

Some miRNAs inhibit the expression of tumor-suppressor genes in normal tissues, elucidating the reason for miRNAs acting as oncogenes. Chu *et al* found that miR-590-5p acts as an oncogene by targeting the *CHL1* gene and promotes cervical cancer proliferation (40). Ma *et al* also found that miR-34a targets GAS1 to promote cell proliferation and inhibit apoptosis in papillary thyroid carcinoma (41). To predict target genes of miR-130a, we screened 26 targets for miR-130a by prediction tools, and found that 12 target genes were associated with tumors. We focused on RUNX3 and Smad4 since it has been previously shown that RUNX3 and Smad4 as tumor suppressor genes are associated with gastric cancer. RUNX3 has been thought to be the target gene of miR-130a in other tissues. It has been shown that miR-130a directly inhibited the expression of the tumor-suppressor gene *RUNX3*, which resulted in the activation of Wnt/β-catenin signaling and increased drug resistance (23). miR-130a also contributes to endothelial progenitor cell dysfunction by targeting RUNX3 (42).

In the present study, Smad4 was excluded since the inhibited expression of miR-130a did not increase Smad4 expression, although RUNX3 expression was significantly increased following transfection with anti-miR-130a. miR-130a expression was inversely correlated with that of RUNX3 in gastric cancer tissues. The result shows that miR-130a attenuates RUNX3 expression in gastric cancer tissues. Thus, RUNX3 may be a target gene of miR-130a. Luciferase reporter assays were performed to confirm miR-130a directly targets RUNX3, and we found that miR-130a directly interacts with RUNX3 by binding to RUNX3 3′UTR. Thus, miR-130a can directly target tumor-suppressor gene *RUNX3*. Furthermore we found that miR-130a significantly increases gastric cancer cell migration, invasion and proliferation, which was completely restored after RUNX3 restoration. The results show that miR-130 promotes gastric cancer tumorigenic abilities by targeting RUNX3, and it elucidates the reason for miR-130a acting as an oncogene in gastric cancer.

In conclusion, the results show that miR-130a expression was increased in gastric cancer and was associated with the overall survival of gastric cancer. miR-130a can be a potential circulating diagnostic biomarker for gastric cancer. Thus, miR-130a may be used as a biomarker for gastric cancer diagnosis and prognosis. Furthermore, miR-130a acts as an oncogene that promotes gastric cancer tumorigenesis by targeting the tumor-suppressor gene *RUNX3*. Thus, future studies on the anticancer mechanisms of miR-130a may contribute to the development of new therapeutic strategies for gastric cancer.

## Figures and Tables

**Table I:** Clinicopathological characteristics of the patient cohort.

Characteristics	Total cases (n=41)
Age (years)	
Range	33–88
Mean	54
Median	57
Pathological T (%)	
PT0-T_is_	12 (29.2)
PT_1_	9 (22.0)
PT_2_	6 (14.6)
PT_3_	8 (19.5)
PT_4_	6 (14.7)
Lymph node metastases (%)	
PN_0_	16 (39.0)
PN_1–3_	25 (61.0)
Dead/alive (%)	
Dead	25 (61.0)
Alive	16 (39.0)

**Table II:** Area under the ROC curve analysis of CEA, CA-199 and miR-130a expression in the serum samples of the gastric cancer patients and controls.

Variables	Area	SE^a^	Asymptotic significance^b^	Asymptotic 95% CI	
				Lower bound	Upper bound
CA-199	0.750	0.054	0.000	0.644	0.855
CEA	0.749	0.053	0.000	0.644	0.853
miR-130a	0.870	0.040	0.000	0.792	0.948

Statistics may be biased.

a Under the non-parametric assumption;

b null hypothesis, true area, 0.5. SE, standard error. CI, confidence interval; ROC, receiver-operating characteristic; CEA, carcinoembryonic antigen.

**Table III:** miR-130a predicted targets.

Total target genes	Target genes associated with tumors
*CTSA*, *STK38L*, *ZFPM2*, *BMPR2*, *ACVR1*, *LDLR*, *DNM2*, *ATG2B*, *DICER1*, *PPARG*, *KLF4*, *HOXA10*, *CSF1*, *MEOX2*, *APP*, *ATXN1*, *TAC1*, *HoxA5*, *MAFB*, *ESR1*, *IFITM1*, *RUNX3*, *MET*, *RAB5A*, *Smad4*, *Smad5*	*HoxA5*, *MAFB*, *ESR1*, *IFITM1*, *RUNX3*, *MET*, *RAB5A*, *Smad4*, *Smad5*, *ATG2B*, *KLF4*, *HOXA10*

RUNX3, runt-related transcription factor 3.
